# Detection of Targetable Genetic Abnormalities in Neuroblastoma Circulating Tumour DNA

**DOI:** 10.3390/ijms26199466

**Published:** 2025-09-27

**Authors:** Marina Danilenko, Sharanya Nath, Jack Baines, Freya Gordon, Swathi Merugu, Lisa M. Allinson, Aaron Potts, Bethany Collins, Angharad Goodman, Samuel E. Kidman, Ciaron McAnulty, David Jamieson, Deborah A. Tweddle

**Affiliations:** 1 Wolfson Childhood Cancer Research Centre, Translational & Clinical Research Institute, Newcastle University Centre for Cancer, Herschel Building, Newcastle upon Tyne NE1 7RU, UK; marina.danilenko2@newcastle.ac.uk (M.D.); snath7@wisc.edu (S.N.); freya.gordon.24@ucl.ac.uk (F.G.); merugus@mskcc.org (S.M.); lisa.allinson@newcastle.ac.uk (L.M.A.); 2 Newcastle Genetics Lab., Newcastle upon Tyne Hospitals NHS Foundation Trust, Newcastle upon Tyne NE1 4EP, UK; jack.baines@nhs.net (J.B.); bethany.collins9@nhs.net (B.C.); sam.kidman@nhs.net (S.E.K.); ciaron.mcanulty@nhs.net (C.M.); 3 Division of Hematology, Medical Oncology and Palliative Care, Department of Medicine, School of Medicine and Public Health, University of Wisconsin, Madison, WI 53726, USA; 4 Childhood Brain Tumour Research Group, Developmental Biology and Cancer, Great Ormond Street Institute of Child Health, University College London, London WC1N 1EH, UK; 5 The Immune Monitoring Facility, Ludwig Centre for Cancer Immunotherapy, Memorial Sloan Kettering Cancer Center, New York, NY 10021, USA; 6 Translational & Clinical Research Institute, Newcastle University Centre for Cancer, Paul O’Gorman Building, Newcastle upon Tyne NE2 4HH, UK; david.jamieson@newcastle.ac.uk; 7 Department of Paediatric Oncology, Great North Children’s Hospital, Newcastle NE1 4LP, UK

**Keywords:** circulating tumour DNA, liquid biopsy, next-generation sequencing, ddPCR, paediatric cancer, neuroblastoma

## Abstract

Neuroblastoma (NB) is an aggressive childhood cancer requiring intensive multimodal therapies in high-risk (HRNB) patients. Currently, invasive surgical biopsies are required to classify NB risk group and assign treatment based on the tumour genetic profile. Circulating tumour DNA (ctDNA) obtained from blood samples can be used to identify tumour biomarkers. Here we applied targeted next-generation sequencing (tNGS) using a panel of 42 genes to analyse 32 NB ctDNA samples for the presence of single-nucleotide variants and copy number changes from 28 patients in all NB risk groups. In two additional ctDNA samples, droplet digital PCR was used to detect hotspot *ALK* variants. Pathogenic mutations with a variant allele frequency (VAF) > 1% were identified in 13/32 (41%) ctDNA samples. *ALK* and *PTPN11* were the most frequent, each being detected in 4/32 (13%) samples, together with oncogene amplifications. Targeted NGS of ctDNA detected actionable variants, including those absent in the diagnostic primary tumour due to spatial and temporal heterogeneity. Our findings confirm the usefulness of ctDNA in detecting genetic abnormalities in NB.

## 1. Introduction

Neuroblastoma (NB) is the most frequent extracranial solid malignancy in children below the age of 5 years [1]. Around 100 new cases of NB are diagnosed in the UK each year, and about half of these are high-risk neuroblastoma (HRNB) with a 5-year event-free survival (EFS) below 50%.

Diagnosis and risk group assignment for NB requires an invasive tumour biopsy, which may be associated with considerable morbidity, particularly in patients below the age of 18 months [2]. An alternative non-invasive means of performing genetic studies in patients with solid tumours is through liquid biopsy sampling to assess circulating tumour cells and circulating tumour DNA (ctDNA) [3,4]. Liquid biopsies are not only safer in young patients but also provide knowledge of intra-tumoural heterogeneity [4].

Cell-free DNA (cfDNA) includes circulating short fragments of double-stranded DNA found in peripheral blood. Circulating tumour DNA (ctDNA) is the fraction of cfDNA originating from tumour tissue in cancer patients and generally constitutes less than 0.1–10% of the total cfDNA present in peripheral blood [5]. Recent studies of NB [6,7,8] and other cancers [9,10] have established that genome-wide DNA sequencing from blood plasma is a promising method for assessing tumour burden. These studies demonstrate, that in metastatic cancer, ctDNA in plasma reflects a composite genetic profile of all existing tumours in a patient. The half-life of ctDNA ranges from 16 to 150 min [10] and helps to record real time intra-tumoural events.

ctDNA analyses offer the potential to characterise NB tumour biology, which is not possible by studying the primary tumour alone. The purpose of this study was to detect and characterise the genetic abnormalities in NB ctDNA collected from patients across different risk groups using targeted next-generation sequencing (tNGS), and in a subset droplet digital PCR (ddPCR), and to compare the findings with matched primary tumour data where available.

## 2. Results

### 2.1. Pathogenic Variants Identified Following ctDNA Variant Filtering

On average, the pipeline called *n* = 120 raw variants per sample. After stringent filtering (see Section 4), 12/32 ctDNA samples had 15 driver variants in total (Table 1). Among these, five were pathogenic *ALK* variants, three were pathogenic *TP53* variants and four pathogenic *PTPN11* variants (Table 1). In total, 10/15 identified ctDNA variants were predicted by in silico tools to have a detrimental impact on protein function and were recorded in the COSMIC database (Table 1) [11]. The remaining variants were classified either as ‘likely pathogenic’ or ‘variants of uncertain significance’ (VUSs), as all of them had low ‘rare exome variant ensemble learner’ (REVEL) [12] scores (≤0.7) and were not reported in the literature and/or had no COSMIC presence. Finally, two out of three germline DNA samples and two out of four cell-line control DNA samples had driver variants. Previously reported findings were confirmed in the cell lines (Table 1).

### 2.2. A High Proportion of Variants Were Identified in NB ctDNA but Not in NB Tumours

**Figure 1:**
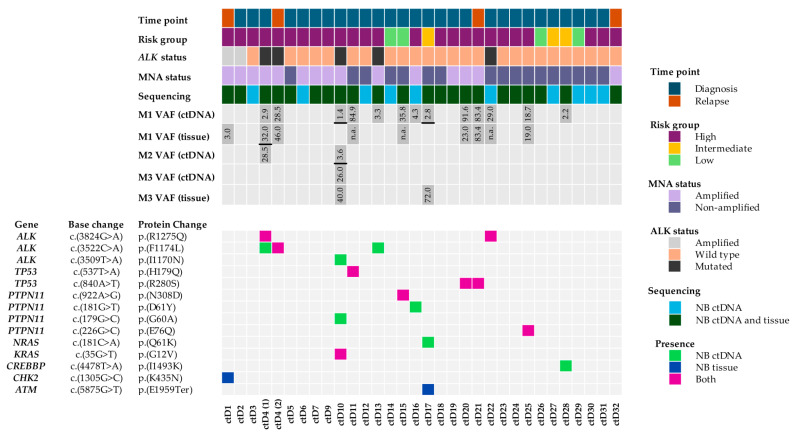
Oncoplot showing comparison of sequencing data between matched NB ctDNA and NB tumours. ctDNA (1–32) is displayed on the *x*-axes. ctD8 was excluded from further analysis, as the cfDNA concentration was below the threshold value of 20%. Information about patient cohort, VAFs (variant allelic frequencies) and the identified variants are displayed on the *y*-axes. Three samples had more than one mutation, and hence there are VAFs for M1, M2 and M3 displayed (with M1 being the top one, M2 the middle one and M3 the bottom one on the oncoplot). VAFs from different variants are separated by black lines. ctD4 is included twice, as an additional relapse sample was obtained from this patient and assessed by ddPCR. Samples 20 and 21 are paired diagnosis and relapse samples from the same patient.

Twenty-two ctDNA samples had matching tumour NGS data available, and thus 16 variants could be compared between liquid biopsies and NB tumours (Figure 1). Strikingly, only nine variants were identified in both ctDNA and matching tumours. The other seven variants were either present in ctDNA only (five mutations: two in *ALK*, one in *PTPN11*, one in *NRAS* and one in *CREBBP*) or only identified in the NB tumours (one mutation in *ATM* and one in *CHK2*) (Figure 1).

### 2.3. Spatial and Temporal Heterogeneity of Heterozygous ALK Mutations

**Figure 2:**
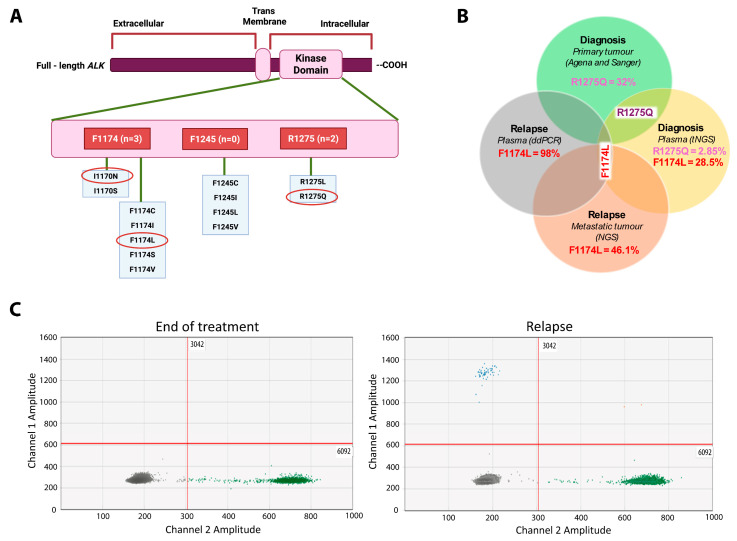
Spatial and temporal heterogeneity of *ALK* variants. (**A**) Distribution of pathogenic *ALK* positions across the tyrosine kinase (TKD) domain within ctDNA. Numbers in red rectangles reflect three cases identified with *ALK* F1174 mutations, no cases with an *ALK* F1245 mutation and two cases with an *ALK* R1275 mutation. (**B**) Venn diagram showing the spatial and temporal heterogeneity of activating *ALK* variants in the tumour and plasma obtained from patient ctD4. (**C**) *ALK* ddPCR on ctDNA samples collected at the end of treatment when the patient was in remission and 3 months later at relapse. Grey droplets are those with no DNA attached, while green droplets represent the wild-type *ALK* allele. F1174L variant (visualised as blue droplets) was not detected at the end of treatment but was identified at relapse.

*ALK* was found to be the most commonly mutated gene in this study. A total of five driver *ALK* variants were observed in four ctDNA samples (Figure 1). Three hotspot kinase domain mutations (R1275Q, F1174L and I1170N) were observed (Figure 2A). R1275Q was present in two ctDNA samples (ctD4 and ctD22), and F1174L was present in two ctDNA samples (ctD4 and ctD13). I1170N was present in one ctDNA sample (ctD10) (Table 1).

The most striking observation to emerge from the *ALK* variant data was from sample ctD4. The Venn diagram (Figure 2B) shows that two variants, encoding R1275Q and F1174L, were detected in the plasma sample from diagnosis, with VAFs of 2.85% and 28.5%, respectively. However, in the diagnostic primary tumour only the R1275Q variant was detected by Sanger sequencing and Agena Mass Array [13] (VAF: 32.0%) (Figure 2B). In a separate plasma sample analysed by ddPCR at the end of treatment, neither R1275Q nor F1174 variants were detected. Figure 2C shows ddPCR results for F1174L at the end of treatment and relapse; results for R1275Q are not included. In a metastatic relapse sample and a plasma relapse sample, 3 months later only the F1174L mutation was detected by targeted NGS from SMPaeds data on the metastatic tumour [14,15] (VAF: 46.1%) and by digital-droplet PCR (ddPCR) (VAF: 98%) on the plasma (Figure 2B,C). R1275Q was not detected in either tumour or plasma at relapse. This patient did not receive an ALK inhibitor at any stage of treatment. This data reflects the presence of spatial and temporal intra-tumoural heterogeneity. It also shows that ctDNA is more representative of malignant clones promoting NB relapse than the primary tumour.

### 2.4. Pathogenic, Homozygous Somatic TP53 Mutations

**Figure 3:**
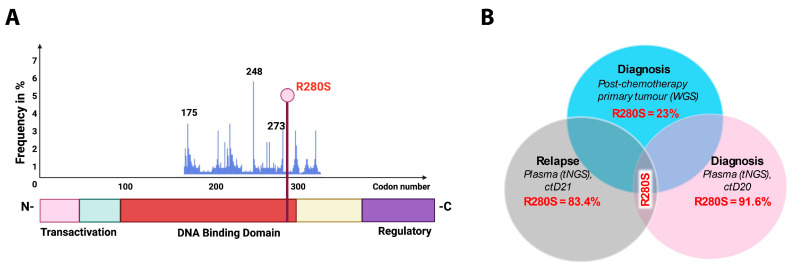
Longitudinal analysis of a homozygous *TP53* variant detected in NB ctDNA and the primary tumour from the same patient. (**A**) Position of the *TP53* variant within the DNA binding domain. (**B**) Venn diagram depicting the *TP53* variant detected across three samples: the diagnostic plasma sample, the post-chemotherapy resected primary tumour and the relapse plasma sample. WGS—whole-genome sequencing.

In this study four *TP53* pathogenic variants with high VAFs were observed in the DNA binding domain of the *TP53* gene (Table 1 and Figure 3A). Three were found in ctDNA and one in a germline DNA sample (Table 1). Observed protein changes were as follows: H179Q in ctD11 and gD1 and R280S in ctD20 and ctD21. Figure 3B shows a homozygous *TP53* variant detected in the diagnosis plasma sample (ctD20), post-chemotherapy primary tumour and relapse plasma (ctD21) samples. The lower VAF for the post-chemotherapy tumour reflects the lower tumour cell content (estimated: 30%, calculated: 19%). Homozygosity was confirmed by 17p loss of heterozygosity (Table A1). In another diagnostic case (ctD11), with a homozygous somatic *TP53* mutation (VAF: 84.9%) caused by concomitant 17p loss (Table A1), the matching diagnostic germline sample had a VAF of 1.4%, suggesting that the gDNA was contaminated with tumour DNA when collected (Table 1).

### 2.5. Heterozygous, Pathogenic PTPN11 Variants

Pathogenic *PTPN11* variants (RAS-MAPK pathway gene family) were observed in five samples (Table 1). A pathogenic germline variant, *PTPN11* N308D, in both ctD15 and gD2 (germline DNA) was identified in a patient with Noonan syndrome and low-risk NB [16,17,18] with VAFs of 35.8% and 43.9%. In the other three cases, activating *PTPN11* mutations were detected within the N terminal domain: G60A (VAF: 3.6%) in the case with a concomitant *KRAS* and *ALK* mutation (ctD10) and in the other two cases at the known hotspots E76Q (VAF: 18%) and D61Y (VAF: 4.34%).

### 2.6. Oncogene Amplifications Detected in ctDNA

**Figure 4:**
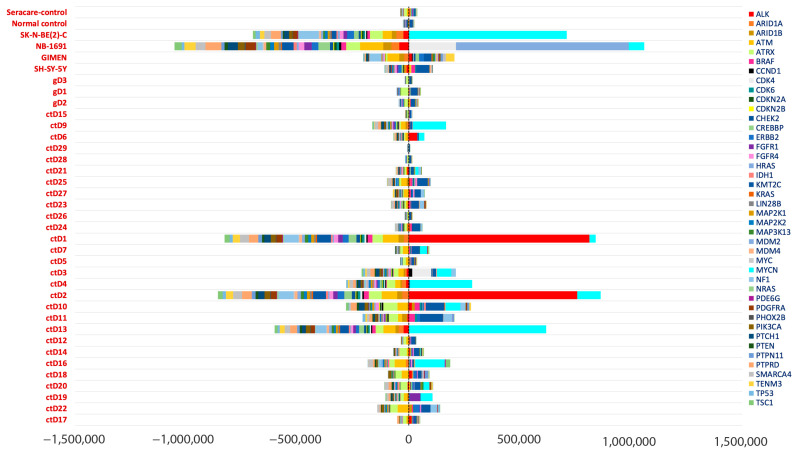
Copy number aberrations detected in NB ctDNA. Bar graph shows inverse z-scores for each target region amplified compared to all other regions in the same sample. The regions with a significant copy number change (amplification or gain) are highlighted with longer bins in the positive quadrant. Numerical coverage data could not be retrieved for ctD30, ctD31 and ctD32, so these samples were omitted. ctD1 and ctD2 and ctD20 and 21 are paired samples at diagnosis and relapse, respectively.

Higher values for coverage were observed in regions corresponding to known oncogene amplification sites (compared to other genes) (Figure 4).

**Figure 5:**
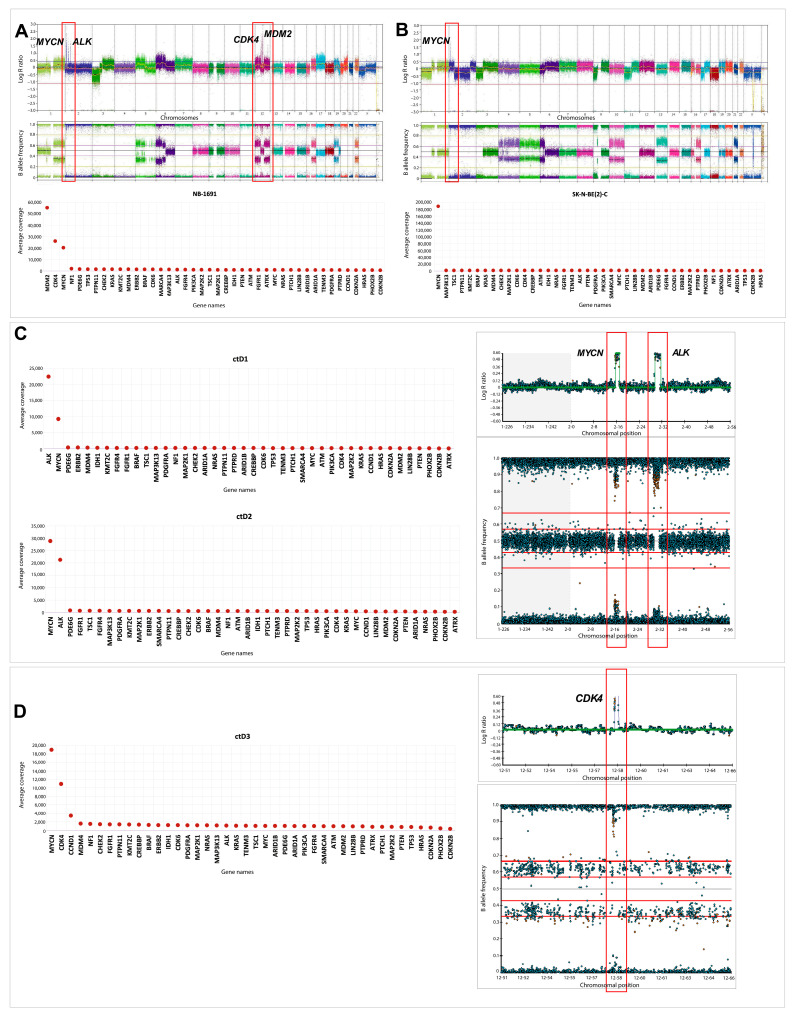
Amplification of *MYCN* (MNA), *ALK* and *MDM2* oncogenes. The 2D line graph depicts the relative coverage of all 42 targeted genes compared to the respective SNP array data from primary tumours or ctDNA. (**A**) NB-1691 cell line: *MYCN*, *CDK4* and *MDM2* amplification, but not *ALK*, were detected by relative coverage analyses compared with the SNP array. (**B**) MNA in SK-N-BE(2)-C cell line. (**C**) *ALK* amplification and MNA detected in ctD1 and ctD2 paired samples. (**D**) MNA, *CDK4* and *CCND1* amplification in ctD3.

*MYCN* amplification was detected in 14/32 ctDNA samples and confirmed in 2 known *MYCN* amplified cell lines (Figure 4 and Figure 5A,B). This was consistent with the amplification status detected by Illumina CytoSNP-850k arrays (Table A1). Furthermore, co-amplification of *ALK* and *MYCN* was observed in the ctDNA samples ctD1 and ctD2, taken from the same patient at diagnosis and relapse, and was consistent with SNP array results in the primary tumour and ctDNA [19] (Figure 5C). *CDK4* amplification, previously associated with a poor prognosis in NB patients [20,21], was detected in ctD3 and confirmed in the NB-1691 cell line (Figure 5A,D). The presence of *MDM2* amplification was also confirmed in the NB-1691 cell line (Figure 5A) and the presence of *CCND1* amplification in ctD3 (Figure 5D). However, neither *ALK* amplification in NB-1691 cells [22] nor *FGFR1* amplification in ctD19, which were evident on SNP arrays (Table A1), were detected in ctDNA (Figure 4 and Figure 5).

## 3. Discussion

The use of liquid biopsy in the management of patients with cancer, either alongside traditional histopathology or as the primary method of obtaining clinically relevant biomarker data, affords clinicians access to real-time tumour monitoring and the ability to detect druggable targets as a tumour evolves. NB frequently metastasises to bone and bone marrow, making liquid biopsy particularly useful for patients with metastatic NB. CfDNA can be extracted from as little as 0.5–2 mL of plasma [23,24,25] and is a viable alternative to invasive biopsies, which increase morbidity in young patients.

Using a targeted gene panel and sequencing ctDNA from plasma samples, we aimed to detect driver events and oncogenic amplifications in ctDNA and to compare these with tumour biopsy samples, where available. Results from this study showed that the bioinformatics pipeline was able to detect all clinically relevant variants, including those with low VAFs (>1%), in ctDNA. Strikingly, 7/16 identified driver ctDNA variants for which tumour NGS data was available, were only present in ctDNA and not in NB tissue. This can be explained by the intra-tumoural heterogeneity of NB when the variants were absent in the biopsied tumour regions while present in ctDNA representing a pool of variants from multiple tumour regions [15,26]. All the variants detected only in ctDNA were at a VAF of <5% and thus likely to be sub-clonal or due to low ctDNA content in the blood. A threshold of 5% has been assigned for most clinical NGS reporting on the bulk tumour, and thus variants with a VAF < 5% could be omitted during data analyses. In two cases, DNA from the NB tumour contained *CHK2* (VAF = 3%) and *ATM* (VAF = 72%) mutations, which were absent in paired ctDNA at the same time point. This could be due to low ctDNA shedding into the blood or variants being sub-clonal. Tumour-only mutations have been previously reported in paired tumour and ctDNA studies and are typically explained by low levels of ctDNA in the patient’s blood [15,26].

Importantly, an *ALK* variant (F1174L) not detected in the diagnostic primary tumour biopsy using the Agena Mass Spectrometer assay, which detects mutations at a VAF of 1.3% [13], was present in diagnostic ctDNA (ctD4), the metastatic relapse biopsy and relapse ctDNA (Figure 2B). This confirms that ctDNA is more reflective of the global genomic variation in a tumour as it overcomes the problem of intra-tumoural heterogeneity missed by a single-site biopsy. In ctD4 two *ALK* mutations were identified in diagnostic ctDNA (R1275Q and F1174L), in line with other ctDNA studies reporting several *ALK* mutations in a single ctDNA sample [15,26,27,28,29]. This case demonstrates both spatial and temporal heterogeneity with different *ALK* mutations detected in the diagnostic primary tumour (R1275Q) and relapsed metastatic tumour (F1174L), as well clonal evolution with loss of the R1275Q mutation between diagnostic and relapse ctDNA, together with an increase in the VAF of the F1174L mutation. The clonal evolution occurred in the absence of selective pressure from patient treatment with an ALK inhibitor.

In keeping with other studies [30,31], pathogenic *ALK* mutations were the commonest mutations in this study, while *PTPN11* mutations were the second most frequent (Table 1 and Figure 1). A study by Ackermann et al. [32] sequenced NB genomes, where they also found *PTPN11* to be commonly mutated. This highlights the importance of developing treatments for *PTPN11* mutated NB, the efficacy of which could be tracked by serial ctDNA monitoring. The Ackermann study reported ALK-RAS pathway mutations to be associated with poor outcomes in all NB risk groups when associated with telomere maintenance mechanisms. In our study, ctD10 harboured two RAS-MAPK (*KRAS*-G12V and *PTPN11*-G60A) mutations and an *ALK*-I1170L mutation, in addition to *MYCN* amplification (a telomere maintenance mechanism) (Table 1). We also detected four ctDNA samples (ctD10, ctD16, ctD20 and ctD21) having RAS-MAPK pathway or *TP53* mutations alongside *MYCN* amplification and hence falling into a very poor prognostic group.

The bioinformatic pipeline was able to detect *MYCN*, *ALK*, *CDK4* and *MDM2* amplifications. However, *ALK* amplification in NB-1691 cells [22] and *FGFR1* amplification in ctD19 were not detected, most likely due to only partial amplification of these oncogenes in regions not covered by the gene panel. Furthermore, the tNGS approach failed to detect other copy number events, as the gene panel was limited to 42 known target genes and did not cover the entire genome (Table A2). Whole-exome sequencing (WES) or low-coverage WGS would be required to detect all copy number changes in these samples

Liquid biopsy-based analysis of ctDNA offers several advantages in the management of NB. Firstly, it is a non-invasive procedure that can be performed longitudinally, allowing for monitoring of disease progression and treatment response over time, as well as early relapse detection. Secondly, it provides a comprehensive assessment of tumour heterogeneity, enabling detection of genetic alterations that may not be captured by single-site tumour biopsies. Thirdly, liquid biopsy can identify actionable variants, guiding personalised treatment decisions.

While our study highlights the potential of ctDNA monitoring in NB, it is limited by its size and the use of mainly diagnostic samples with longitudinal samples in only three cases. The longitudinal testing of plasma samples provides more information about NB progression over time, uncovering possible mechanisms of treatment resistance and relapse, as recently reported by studies of *ALK* mutation tracking in ctDNA collected from NB patients treated with *ALK* inhibitors [26,28,29]. Previous studies reported both reductions in the VAFs of *ALK* mutations in longitudinal ctDNA sampling during successful patient treatment with ALK inhibitors [7,15,26,28,29,33,34] but also the emergence of resistance mutations, including both bypass mutations, particularly of the RAS-MAPK pathway [26,28,29,34], as well as compound *ALK* mutations [29]. Importantly, one study also reported loss of *ALK* mutations in some patients who relapsed during treatment with the third-generation ALK inhibitor lorlatinib, due to selection for *ALK* wild-type clones by lorlatinib treatment [29]. Furthermore, we have previously reported selection for *ALK* wild-type clones in tumour DNA from two patients between diagnosis and relapse in the absence of ALK inhibitor treatment [13]. These studies emphasise the importance of serial ctDNA monitoring for *ALK* mutations in patients with NB, particularly when treating patients with ALK inhibitors.

In conclusion, our study confirms the potential of liquid biopsy, specifically ctDNA analysis, to study NB in a non-invasive way.

## 4. Materials and Methods

### 4.1. Plasma and Cell-Line Samples

Plasma and germline DNA samples were obtained from VIVO biobank (formerly the Children’s Cancer and Leukaemia Group (CCLG) Tissue Bank) (study number 2021 BS 03). The NGS study included *n* = 41 samples, of which *n* = 32 were ctDNA samples from NB patient plasma, including *n* = 3 paired samples (2 paired diagnosis and relapse and 1 paired blood and bone marrow plasma), *n* = 3 germline DNA, *n* = 4 control NB cell-line DNA (SH-SY-5Y, NB-1691, SK-N-BE(2)-C and GIMEN) and *n* = 2 control samples, one a synthetic control (Seraseq ctDNA. Complete AF0.1%, Seracare) (Seracare, Milford, MA, USA) and the other a normal genomic DNA control (Table A1). Two additional plasma samples (from the end of treatment and three months later at relapse) from the ctD4 patient were obtained and used for ddPCR. In total, 28 NB cases were studied: 4 low risk, 3 intermediate risk and 21 high risk (Figure 1 and Table A1).

### 4.2. DNA Extraction

DNA from 4 cell lines and ctDNA from 0.5 mL plasma were extracted using EZ1 Advanced XL (QIAGEN, Hilden, Germany) kits: the EZ1 DNA 200 μL Blood Kit for plasma samples and the EZ1 DNA Tissue Kit for cell-line pellets, according to the manufacturer’s instructions. cfDNA content was assessed using the Agilent TapeStation 4200 (Agilent, Santa Clara, CA, USA with the cell-free DNA ScreenTape assay. Sample ctD8 was excluded from further analysis, as the cfDNA concentration was only 6%, below the threshold value of 20%. The library preparation method also incorporated unique molecular indices (UMIs) ligated to the ends of DNA fragments. The UMI code is a sequence of short nucleotides that uniquely identifies each molecule in a sample library, enabling greater precision during sequencing and error correction. The prepared libraries were quality assessed using Bioanalyzer 2400 High Sensitivity D1000 chip assays (Agilent, Santa Clara, CA, USA) by quantifying fragments between 35 and 1000 bp. The size interval used for library preparation was approximately between 200 and 400 bp, where the cfDNA fragment peak formed. The prepared libraries were also quantified using the Qubit Fluorometer 2.0—double-stranded DNA high-sensitivity assay (ThermoFisher, Waltham, MA, USA).

### 4.3. Library Preparation and Next-Generation Sequencing

Sequencing library preparation for ctDNA samples was performed according to the manufacturer’s instructions using a QIAseq Targeted Custom DNA Panel of 42 genes (Table A2). The gene panel was compiled according to recommendations of the SIOPEN Biology Group [35]. Targeted NGS (Illumina NextSeq 550 System (Illumina, Inc., San Diego, CA, USA)) was performed on *n* = 31 patient cfDNA samples, *n* = 4 cell-line DNA samples, *n* = 3 germline DNA samples and *n* = 2 control DNA samples. Raw reads from the NextSeq550 were aligned and variant calling was performed using CLC Genomics Workbench 22 (CLC Bio, version 22.0, Aarhus, Denmark). An analysis of the UMI coverage per gene was carried out across all samples.

### 4.4. Variant Filtering

The mean read depth was calculated to be 2228x after excluding the read depth of over- and underrepresented amplified genes and genes on the X chromosome, consistent with the manufacturers’ specifications of a mean read depth of 2500x for 20 samples on a mid-output flow cell with the QiaSeq kit (QIAGEN). Variants with coverage < 100× and frequencies < 0.5% were filtered out. CLC Genomics workbench tools software was used to remove sequencing artefacts, PCR duplicates and recurrent variants. The resulting variants were then annotated using the Ensembl Variant Effect Predictor (VEP) [36] with Genome Aggregation Database (gnomAD r2.1, exomes only [37]) population frequencies, ‘Sorting Intolerant from Tolerant’ (SIFT 5.2.2) [38] algorithm scores, ‘Polymorphism Phenotyping v2’ (PolyPhen 2.2.2) [39] scores (range from 0 to 1; 0 = most likely benign, 1 = most likely damaging), ‘rare exome variant ensemble learner’ (REVEL) [12] scores (range from 0 to 1; 0 = likely benign, 1 = likely pathogenic) and COSMIC IDs [11]. Using a standard approach, the pipeline filtered out germline variants by removing the SNPs found in the ‘dbSNP common’ database [40] (dbSNP build 151, with ≥1% minor allele frequency (MAF) present in any of the five super-populations). Further stringent filtering for germline variants was performed manually by annotating the variants with gnomAD frequencies using VEP, excluding variants occurring in different population groups (gnomAD r2.1, exomes-only database) with a frequency of more than 1.0%. Furthermore, germline data was used from WGS from blood samples corresponding to ctD19, ctD20, ctD23, ctD25, ctD26 and ctD28 sequenced at 30x coverage (NHS England Whole Genome Sequencing (WGS) service). In these cases, the final filtered variants generated were subjected to a comparative analysis against the germline variants identified in their matched WGS data.

### 4.5. Coverage Analysis

An analysis of the UMI coverage per gene was carried out across all samples and graphs were plotted. The UMI coverage was assessed using two metrices, relative UMI coverage (average coverage) and UMI read depth, which helped evaluate the reliability of the tNGS panel in covering all the targeted regions. To validate the coverage data to correctly detect gene amplification status, the results, wherever possible, were compared with SNP array data generated from Illumina CytoSNP-850k arrays using Illumina BlueFuse multi v4.4 software or Nexus Copy Number v10.0 software (Bionano Genomics Inc., San Diego, CA, USA).

### 4.6. Classification of Somatic Variants

Classification of filtered variants was determined by considering both the biological and clinical relevance of the data [5]. The potential pathogenicity of variants was determined using SIFT [38], PolyPhen-2 [39], REVEL [12] scores and COSMIC [11] presence. Variants not classified previously in COSMIC or from a literature review were not discarded but retained for further investigation. The list of somatic driver variants was also compared with those obtained from analysis of the primary or metastatic tumours using WGS (*n* = 9) or a different targeted or whole-exome sequencing platform (*n* = 13). This included NGS from Stratified Medicine Paediatrics (SMPaeds) testing [14] or tNGS using a SIOPEN 38 gene panel [35] where available, together with results of *ALK* sequencing using Sanger sequencing or Agena Mass Spectrometer arrays [13] (Figure 1).

### 4.7. ddPCR

High-sensitivity testing for the ALK c.3522C>A p.(F1174L) and c3824G>A p.(R1275Q) variants was performed by droplet digital PCR (ddPCR) using commercially designed primers and probes obtained from Bio-Rad (assay ID numbers: dHsaMDV2516768 ALKp.R1275Q and dHsaMDV2010083ALK p.F1174L c.3522C>A). Cell-free DNA extracted from peripheral blood was amplified by standard PCR methods and fragmented by restriction digest using HaeIII. PCR products were then separated using the Bio-Rad QX-200 ddPCR system following standard Bio-Rad protocols. Analysis was performed using QX Manager Software Standard Edition v2.1 obtained from BioRad (Hercules, CA, USA). The sequence for the *ALK* F1174L c.3522C>A primer is hg19|chr2:29443634-29443756, and for the *ALK* p.R1275Q primer the sequence is hg19|chr2:29432603-29432725.

### 4.8. Statistical Analyses

Target-region coverage analysis was carried out for all samples using the CLC Genomics Workbench tools. The resulting numerical data per targeted region per gene was used to calculate a z-score for the determination of CNVs from coverage data [41].

$$Z=\frac{x-\mu }{\sigma }$$

where x = raw score, μ = mean of data array and σ = standard deviation of the data array.

## Figures and Tables

**Table 1:** Pathogenic variants detected in ctDNA (ctD), germline DNA (gD) and cell-line DNA samples. All variants were identified as missense variants, apart from *NF1*, which was identified as a splice donor. Abbreviations: Del—deleterious, POD—possibly damaging, PRD—probably damaging, B—benign, Chr—chromosomal. REVEL score > 0.7 = pathogenic. 1 = SHSY5Y cell line, 2 = SKNBE(2c) cell line. ^a,b^ = Identifies ctDNA and matched gDNA pairs. ^c^ = Identifies paired samples from the same patient at diagnosis and relapse.

Sample	VAF	Variant Gene	Chr. Location	Base Change	Protein Change	COSMIC ID	SIFT Score	Polyphen	REVEL
								Score	Score
ctD4	2.85	*ALK*	chr2 (p23.2)	c.(3824G>A)	p.(R1275Q)	COSV66555567	Del (0)	PRD (1)	0.885
ctD4	28.5	*ALK*	chr2 (p23.2)	c.(3522C>A)	p.(F1174L)	COSV66556325	Del (0.03)	POD (0.641)	0.752
ctD10	1.38	*ALK*	chr2 (p23.2)	c.(3509T>A)	p.(I1170N)	COSV66589132	Del (0)	PRD (1)	0.906
ctD10	26.16	*KRAS*	chr12 (p12.1)	c.(35G>T)	p.(G12V)	COSV55497419	Del (0)	PRD (0.972)	0.91
ctD10	3.61	*PTPN11*	chr12 (q24.13)	c.(179G>C)	p.(G60A)	COSV61006397	Del (0.01)	PRD (0.953)	0.907
^a^ ctD11	84.88	*TP53*	chr17 (p13.1)	c.(537T>A)	p.(H179G)	COSV52673406	Del (0)	PRD (0.98)	0.79
^a^ gD1	1.4	*TP53*	chr17 (p13.1)	c.(537T>A)	p.(H179Q)	COSV52669519	Del (0)	PRD (0.98)	0.79
ctD13	3.33	*ALK*	chr2 (p23.2)	c.(3522C>A)	p.(F1174L)	COSV66556325	Del (0.03)	POD (0.641)	0.752
^b^ ctD15	35.8	*PTPN11*	chr12 (q24.13)	c.(922A>G)	p.(N308D)	COSV61006575	Del (0.03)	B (0.134)	0.838
^b^ gD2	43.9	*PTPN11*	chr12 (q24.13)	c.(922A>G)	p.(N308D)	COSV61006575	Del (0.03)	B (0.134)	0.838
ctD16	4.34	*PTPN11*	chr12 (q24.13)	c.(181G>T)	p.(D61Y)	COSV61004841	Del (0)	PRD (0.997)	0.933
ctD17	2.26	*NRAS*	chr1 (p13.2)	c.(181C>A)	p.(Q61K)	COSV54736310	Del (0.01)	POD (0.709)	N/A
^c^ ctD20	91.64	*TP53*	chr17 (p13.1)	c.(840A>T)	p.(R280S)	COSV52782181	Del (0.03)	POD (0.843)	0.878
^c^ ctD21	83.4	*TP53*	chr17 (p13.1)	c.(840A>T)	p.(R280S)	COSV52801834	Del (0.03)	POD (0.843)	0.878
ctD22	29.03	*ALK*	chr2 (p23.2)	c.(3824G>A)	p.(R1275Q)	COSV66555567	Del (0)	PRD (1)	0.885
ctD25	18.7	*PTPN11*	chr12 (q24.13)	c.(226G>C)	p.(E76Q)	COSV61004751	Del (0.01)	PRD (0.979)	0.733
ctD28	2.2	*CREBBP*	chr16 (p13.3)	c.(4478T>A)	p.(I1493K)	COSV52129182	Del (0)	PRD (0.991)	0.929
1	44.4	*KRAS*	chr12 (p12.1)	c.(35G>T)	p.(G12V)	COSV55497419	Del (0)	PRD (0.972)	0.91
1	50.9	*SMARCA4*	chr19 (p13.2)	c.(2917C>T)	p.(R973W)	COSV60787034	Del (0)	PRD (1)	0.86
1	49.6	*ALK*	chr2 (p23.2)	c.(3522C>A)	p.(F1174L)	COSV66555460	Del (0.03)	POD (0.641)	0.752
2	99.1	*TP53*	chr17 (p13.1)	c.(404G>T)	p.(C135F)	COSV52680475	Del (0)	PRD (1)	0.96
2	100	*NF1*	chr17 (q11.2)	c.(1989_2001del)	p.(G663fs)	N/A	N/A	N/A	N/A

## Data Availability

Data is contained within the article. Sequencing data has been deposited in NCBI SRA (BioProject accession number PRJNA928778).

## Appendix A

### Appendix A.1

**Table A1:** Samples used in the ctDNA study with existing SNP array data from the primary tumour. ctD8 was excluded from further analysis, as the cfDNA concentration was below the threshold value of 20%. ^a,b,c^ = paired ctDNA samples; ^a,b^ were obtained from the same patient at diagnosis and relapse and ^c^ was obtained from ctDNA from the same patient from blood and bone marrow at diagnosis. ^d,e,f^ = ctDNA and matched gDNA pairs. Abbreviations and symbols: NB—Neuroblastoma; Int—Intermediate risk; Int ** = localised, non-MNA, >18 months of age, unfavourable histology; PB—Peripheral blood; BM—Bone marrow; R—Relapse; D—Diagnosis; * denotes cases where SNP array performed on ctDNA [19]; CN-LOH = copy neutral loss of heterozygosity; MNA—*MYCN* amplified; non-MNA—Non-*MYCN* amplified.

Sample Name	Sample Type	NB Risk Group and *MYCN* Status	Time Point	SNP Array Data	
				Gain	Loss
^a^ ctD1	Plasma (PB)	High MNA	R	* 1q, 17q, 18q. *ALK* amplification	* 1p, 5p, 18p.
^a^ ctD2	Plasma (PB)	High MNA	D	*ALK* amplification 11q, 17q.	1p, 10q.
ctD3	Plasma (PB)	High MNA	D	1q13.1q32.1, 2p16.1p25.3, *CDK4* amplification, *CCND1* amplification 6p21.3q27, 12p13.3q24.3, 14q11.2q32.12.	X, 11, 9, 10, 4, 5p.
ctD4	Plasma (PB)	High MNA	D	17q.	1p, 3p, 10, 15q.
ctD5	Plasma (PB)	High Non-MNA	D	11pq, 17q, 18.	11q.
ctD6	Plasma (PB)	High MNA	D	2p23.1(*ALK*), 17q.	1p, 9, 11.
ctD7	Plasma (PB)	High MNA	D	17q22q25.3.	1p31.2p36.33.
ctD9	Plasma (PB)	High MNA	D	16q, 17q, 21.	1p, 3, 4q, 5pq, 6, 8, 14, 15q, 16q, 17p,
					CN-LOH: 9, 10, 11, 19, X.
ctD10	Plasma (PB)	High MNA	D	17p12p11.2, 17q12q22.	3, 5, 4q, 9, 10, 11, 13q, 21q.
^d^ ctD11	Plasma (PB)	High Non-MNA	D	6p, 7p, 13q, 21 & 12.	6p, 6q, 8p, 8q, 11, 17p.
ctD12	Plasma (PB)	High Non-MNA	D	2p, 6pq, 7p, 11p, 12q, 16q, 17q, 22q, 13 & 18.	9p, 11q.
ctD13	Plasma (PB)	High MNA	D	17q.	1p, 3p.
ctD14	Plasma (PB)	Low Non-MNA	D	Whole-chromosome abnormalities only	
^e^ ctD15	Plasma (PB)	Low Non-MNA	D	Whole-chromosome abnormalities only	
ctD16	Plasma (PB)	High MNA	D	1q, 2p, 9q, 11q, 17q, 18p.	1p, 3p, 11q, X.
^f^ ctD17	Plasma (PB)	Int ** Non-MNA	D	1q, 2p, 5p, 11q, 16q, 17q, 20q.	3p, 5q, 6p, 6q, 9p (*PTPRD*), 9q, Y.
ctD18	Plasma (PB)	High Non-MNA	D	1q, 2p (*MYCN)*, 12q, 17q,	3p, 3q, 11q, 19q,
				20q.	22q, Xq21.1 (*ATRX*).
ctD19	Plasma (PB)	High MNA	D	2, 4, 7, 8, 8p11.22p11.21, 12, 15, 17p11.2q25.3, 18, 20, 21, 22, *FGFR1* amplification.	10
^b^ ctD20	Plasma (PB)	High MNA	D	3, 11, 14, 20q13.2q13.33, 21, 22.	1, 2, 8p23.3q21.3, 9, 10p15.3q21.1, 13, 16q11.2q24.3, 18, 17p.
^b^ ctD21	Plasma (PB)	High MNA	R	3, 11, 14, 20q13 2q13.33, 21, 22.	1, 2, 8p23, 3q21.3, 9, 10p15, 3q21.1, 13, 16q11, 2q24.3, 18, 17p.
ctD22	Plasma (PB)	High Non-MNA	D	1q21.1q42.2,6, 7, 8p23.3p22,10p15.3q11.21, 13, 17, 18, 20, 21.	1q42.2q44, 3, 9p24.1 (*PTPRD*), 11, 11q14.1q22.3, 15q13.2q26.3, 19.
ctD23	Plasma (PB)	High Non-MNA	D	4p16, 3q21.23, 7, 13q11q31.3, 13q32, 1q34, 17, 18.	1q44, 2q44, 2q21, 3q37.3, 3, 5q15q35.3, 9p24, 3p22.3, 9p21, 3q34.3, 11, 14, 16, 19, 22, X, LOH: 2q21.3q37.3, 5q15q35.3, 11, 16, 22.
ctD24	Plasma (PB)	High Non-MNA	D	6p, 7pq, 13q, 17pq, 18, 21.	6q, 7p, 17p.
ctD25	Plasma (PB)	High Non-MNA	D	1q, 3q, 6p, 3q, 7, 11q, 1q, 12, 16q,17q, 18.	3p26, 3p12.2, 3p12, 2q29, 4, 6q22, 33q27, 8, 11q13, 3q25, 14, 17p13, 3q21.31,
					19p13, 3p13.11.
ctD26	Plasma (PB)	Low Non-MNA	D	1, 2, 7, 12, 17.	No loss
ctD27	Plasma (PB)	Int Non-MNA	D	2, 5, 7, 13, 17, 22, X.	3, 4, 11, 14, 21 LOH: 3, 14.
ctD28	Plasma (PB)	Int ** Non-MNA	D	Tetraploid.	3, 4, 14, 15, 16.
ctD29	Plasma (PB)	Low Non-MNA	D	No gains.	2, 3, 4, 11, 12, 14, 15, 16, 19, 21, X
					CN-LOH: 11.
^c^ ctD30	Plasma (BM)	High Non-MNA	D	* 2p, 6p, 7, 17q, 18.	3q, 5, 6q,
					9p, 10, 11q, 17p, 19.
^c^ ctD31	Plasma (PB)	High Non-MNA	D	* 2p, 6p, 7, 17q, 18.	3q, 5, 6q,
					9p, 10, 11q, 17p, 19.
ctD32	Plasma (PB)	High MNA	R	* 1q, 2p, 11q, 17q,19q, 3, 5, 6, 7, 8, 12, 13, 14, 16, 20, 21.	* 1p, 11q, 19p, 22q.
^d^ gD1	DNA	High	D	-	-
^e^ gD2	DNA	Low	D	-	-
^f^ gD3	DNA	Int **	D	-	-
SH-SY-5Y	DNA	High Non-MNA	R	1q, 7, 17q.	14p, 22q.
GIMEN	DNA	High Non-MNA	R	2p.	1p, 6q, 11q.
NB-1691	DNA	High MNA	R	*ALK, CDK4, MDM2* amp 3q, 5p, 6p, 12, 17.	3p.
SK-N-BE-(2)C	DNA	High MNA	R	2p.	1p, 17, 18, X, 11p, 9p, 3p.
Control1	Synthetic plasma	-	-	-	-
Control2	Normal control DNA	-	-	-	-

### Appendix A.2

**Table A2:** Gene list for the 42-gene QIASeq custom tNGS panel used in this study.

Variant Gene	Chromosomal Location	Transcript	Function
*ALK*	2p23.1	NM_004304.4	Tyrosine kinase
*ARID1A*	1p36.11	NM_006015.4	Chromatin remodelling factor
*ARID1B*	6q25.3	NM_020732.3	Chromatin remodelling factor
*ATM*	11q22.3	NM_000051.3	Serine/threonine kinase
*ATRX*	Xq21.1	NM_000489.3	Chromatin remodelling factor
*BRAF*	7q34	NM_004333.4	Protein kinase
*CCND1*	11q13.3	NM_053056.2	Cyclin dependent protein kinase
*CDK4*	12q14.1	NM_000075.3	Cyclin dependent protein kinase
*CDK6*	7q21.2	NM_001259.6	Cyclin-dependent protein kinase
*CDKN2A*	9p21.3	NM_001195132.1	Cyclin-dependent protein kinase
*CDKN2B*	9p21.3	NM_004936.3	Cyclin-dependent protein kinase
*CHEK2*	22q22.1	NM_007194.4	Protein kinase
*CREBBP*	16p13.3	NM_004380.3	Acetyltransferase
*ERBB2*	17q12	NM_004448.2	Tyrosine kinase
*FGFR1*	8p11.23	NM_001174067.1	Tyrosine kinase growth factor
*FGFR4*	5q35.2	NM_002011.3	Growth factor receptor tyrosine kinase
*HRAS*	11p15.5	NM_005343.2	GTPase
*IDH1*	2q34	NM_005896.2	Catalytic isozyme
*KMT2C*	7q36.1	NM_170606.3	Histone methyltransferase
*KRAS*	12p12.1	NM_033360.2	GTPase
*LIN28B*	6q16-6q21	NM_001004317.3	RNA-binding protein
*MAP2K1*	15q22.31	NM_002755.3	MAP kinase
*MAP2K2*	19p13.3	NM_030662.3	Tyrosine kinase
*MAP3K13*	3q27.3	NM_001242314.1	Serine/threonine kinase
*MDM2*	12q15	NM_002392.4	TP53 regulator (negative)
*MDM4*	1q32.1	NM_002393.4	TP53 regulator (negative)
*MYC*	8q24.21	NM_002467.4	Transcription factor
*MYCN*	2p24.3	NM_005378.4	Transcription factor
*NF1*	17q11.2	NM_001042492.2	Tumour suppressor
*NRAS*	1p13.2	NM_002524.4	GTPase
*PDE6G*	17q25.3	NM_002602.3	Phosphodiesterase
*PDGFRA*	4q12	NM_006206.4	Tyrosine kinase
*PHOX2B*	4p13	NM_003924.3	Transcription factor
*PIK3CA*	3q26.32	NM_006218.2	Lipid kinase
*PTCH1*	9q22.32	NM_000264.3	Receptor for Hedgehog genes
*PTEN*	10q23.31	NM_000314.4	Phosphatase
*PTPN11*	12q24.13	NM_002834.3	Protein tyrosine phosphatase
*PTPRD*	9p23	NM_002839.3	Tyrosine phosphatase
*SMARCA4*	19p13.2	NM_003072.5	Chromatin regulator
*TENM3*	4q34.3	NM_001080477.1	Neuronal development protein
*TP53*	17p13.1	NM_000546.6	Tumour suppressor
*TSC1*	5q32-q33	NM_000368.5	Tumour suppressor
